# HMGB1 Contributes to the Expression of P-Glycoprotein in Mouse Epileptic Brain through Toll-Like Receptor 4 and Receptor for Advanced Glycation End Products

**DOI:** 10.1371/journal.pone.0140918

**Published:** 2015-10-20

**Authors:** Yan Chen, Xian-Jing Huang, Nian Yu, Yuan Xie, Kang Zhang, Fang Wen, Hao Liu, Qing Di

**Affiliations:** 1 Department of Neurology, Nanjing Brain Hospital affiliated to Nanjing Medical University, Nanjing, Jiangsu, China; 2 Department of Neurology, University of Pittsburgh School of Medicine, Pittsburgh, Pennsylvania, United States of America; Indian Institute of Integrative Medicine, INDIA

## Abstract

The objective of the present study was to investigate the role of high-mobility group box-1 (HMGB1) in the seizure-induced P-glycoprotein (P-gp) overexpression and the underlying mechanism. Kainic acid (KA)-induced mouse seizure model was used for *in vivo* experiments. Male C57BL/6 mice were divided into four groups: normal saline control (NS) group, KA-induced epileptic seizure (EP) group, and EP group pretreated with HMGB1 (EP+HMGB1 group) or BoxA (HMGB1 antagonist, EP+BoxA group). Compared to the NS group, increased levels of HMGB1 and P-gp in the brain were observed in the EP group. Injection of HMGB1 before the induction of KA further increased the expression of P-gp while pre-treatment with BoxA abolished this up-regulation. Next, the regulatory role of HMGB1 and its potential involved signal pathways were investigated in mouse microvascular endothelial bEnd.3 cells *in vitro*. Cells were treated with HMGB1, HMGB1 plus lipopolysaccharide from *Rhodobacter sphaeroides* (LPS-RS) [toll-like receptor 4 (TLR4) antagonist], HMGB1 plus FPS-ZM1 [receptor for advanced glycation end products (RAGE) inhibitor], HMGB1 plus SN50 [nuclear factor-kappa B (NF-κB) inhibitor], or vehicle. Treatment with HMGB1 increased the expression levels of P-gp, TLR4, RAGE and the activation of NF-κB in bEnd.3 cells. These effects were inhibited by the pre-treatment with either LPS-RS or FPS-ZM1, and were abolished by the pre-treatment of SN50 or a combination treatment of both LPS-RS and FPS-ZM1. Luciferase reporter assays showed that exogenous expression of NF-κB p65 increased the promoter activity of *multidrug resistance 1a* (P-gp-encoding gene) in endothelial cells. These data indicate that HMGB1 contributes to the overexpression of P-gp in mouse epileptic brain tissues via activation of TLR4/RAGE receptors and the downstream transcription factor NF-κB in brain microvascular endothelial cells.

## Introduction

Epilepsy is a chronic and devastating neurological disorder characterized by recurrent unprovoked seizures. A substantial proportion (~30%) of patients with epilepsy is refractory to carefully optimized pharmacological treatment [1]. The overexpression of P-glycoprotein (P-gp) induced by seizure activity [2, 3] has been considered to play an important role in the development of drug-refractory epilepsy [4, 5]. However, the precise mechanism underlying the seizure-induced overexpression of P-gp remains elusive [6].

P-gp is an efflux transporter protein encoded by *multidrug resistance1* (*MDR1*) in humans and *mdr1a* (mainly expressed in brain vascular endothelium) and *mdr1b* (mainly expressed in brain parenchyma) in rodents [7, 8]. It has been documented that the increased level and activity of P-gp on the blood-brain barrier (BBB) were associated with the inflammatory process in epileptic brain. Bauer et al. [9] reported that the level of expression of P-gp was increased by extracellular glutamate through N-methyl-D-aspartate (NMDA)/cyclooxygenase-2 (COX-2) pathway. Inflammatory mediator tumor necrosis factor-alpha (TNF-α) was also reported to enhance the activity of P-gp in BBB [10]. Recently, Yin et al. [11] reported that extracellular inflammatory molecule high-mobility group box-1 (HMGB1) may promote drug resistance by upregulating the expression of P-gp in human gastric adenocarcinoma cells. HMGB1-mediated inflammatory pathways have been verified to be activated in many seizure animal models and could initiate and expand inflammation in epileptic tissue [12–14]. The increase of HMGB1 in epileptic brain was observed between 1 h and 3 h after the onset of seizures [15], and the progressive up-regulation of P-gp often occurred at 3–24 h after kainic acid (KA)-induced seizures [16, 17]. Taking together, we hypothesize that HMGB1 may be responsible for the upregulated expression of P-gp in the epileptic brain.

HMGB1, a nuclear chromatin protein, is ubiquitously expressed in all cells, and it obtains a new identity to act as a damage-associated molecular pattern (DAMP) when placed extracellularly [18]. During the pathogenesis of a number of inflammatory, autoimmune diseases and cancers, HMGB1 could play multiple roles and mediate processes ranging from inflammation to repair as well as drug resistance [19]. Toll-like receptor 4 (TLR4) and receptor for advanced glycation end products (RAGE) are both the best characterized receptors identified for HMGB1. Additionally, the two receptors are constitutively expressed by many cell types, and they can be rapidly upregulated upon interaction with their ligands. TLR4 is a member of TLRs, a group of innate immune system receptors that react to pathogen-associated molecular patterns and DAMPs, and mediate many cell responses including inflammation, innate and adaptive immune responses [20]. Activation of TLR4 by HMGB1 in neurons and astrocytes has been proposed as a critical event for decreasing seizure threshold and initiating brain inflammation [15]. RAGE, like TLR4, is a transmembrane receptor playing key roles in innate immunity activation and inflammatory processes [21]. Iori et al. [22] have suggested that RAGE induced in neurons, astrocytes and microvessels by epileptic activity contributes to hyperexcitability underlying seizures, as well as to the proictogenic effects of HMGB1. Nuclear factor-kappa B (NF-κB), a pivotal regulator of immune and inflammatory response, is one of the most important downstream transduction molecules in both the TLR4 and RAGE signaling pathway [20, 21]. In cytosol, NF-κB presents as an inactive form due to the combination of inhibitory kappa B (IκB) to the active subunits P50 and P65 [23]. Once activated, NF-κB can translocate to the nucleus and bind to the promoter region of target genes, including previously reported rat *mdr1* gene, therefore regulating the expression of these genes [24]. Emerging evidence suggests that NF-κB is activated during the neuropathological processes of seizure and epilepsy [25, 26].

In the present study, the effect of HMGB1 on the expression of P-gp was investigated both *in vivo* using a KA-induced mouse seizure model and *in vitro* using a mouse microvascular endothelial cell line, bEnd.3. TLR4 antagonist, lipopolysaccharide from *Rhodobacter sphaeroides* (LPS-RS) [27], RAGE inhibitor, FPS-ZM1 [28], and NF-κB inhibitor, SN50 (amino acid sequence AAVALLPAVLLALLAPVQRKRQKLMP) [29], were used to investigate the involvement of different signal pathways. Our results indicate that HMGB1 increases the expression of P-gp in mouse epileptic brain tissues via activation of TLR4, RAGE, and the downstream NF-κB pathway in brain microvascular endothelial cells. These data provide an insight into the involvement of HMGB1 in seizure-induced overexpression of P-gp.

## Materials and Methods

### 2.1 Animals and ethics statement

Adult male C57BL/6 mice weighing 20±2 g were purchased from Slack Shanghai Laboratory Animals (Shanghai, China) [License Number: 2013–0005]. Following arrival, animals were housed for 2 weeks in a room with a fixed 12 h light-dark cycle and a constant temperature (20±2°C) and humidity (50–60%). All procedures conformed to the international Guide for the Care and Use of Laboratory Animals [30] and were approved by the Biological Research Ethics Committee of Nanjing Medical University (Permit Number: 20110354). All surgery was performed under chloral hydrate anesthesia, and all efforts were made to minimize the suffering.

### 2.2 KA-induced mouse epileptic seizure model and treatment with HMGB1

Forty-seven mice were randomly divided into four groups, (1) normal saline control group (NS group, n = 11), (2) KA-induced epileptic seizure group (EP group, n = 12), (3) EP group pretreated with recombinant human HMGB1 (EP+HMGB1 group, n = 12), and (4) EP group pretreated with BoxA (a fragment of HMGB1 with antagonistic activity; EP+BoxA group, n = 12). For the mice in EP, EP+HMGB1, and EP+BoxA groups, seizure was induced by the stereotactic injection of KA (7 ng in 0.5 μL; Sigma, Louis, MO) into the models’ left hippocampus area (1.8 mm posterior to the bregma, 1.5 mm left lateral from the midline, and 2.0 mm deep from the dura) according to the atlas of Franklin and Paxinos [31]. KA was dissolved in 0.1 M phosphate-buffered saline (PBS, pH 7.4). This dose of KA induces electroencephalographic epileptiform activity in the hippocampus in 100% of injected mice without any mortality [15, 22, 32]. For mice in the NS group, an equal amount of NS was microinjected. At 15 min prior to microinjection with KA, mice in the EP+HMGB1 and EP+BoxA groups were pretreated with HMGB1 (5.5 μg in 1 μL, dissolved in sterile 0.1 M PBS) or BoxA (2.5 μg in 1 μL, dissolved in sterile 0.1 M PBS; HMGBiotech, Milan, Italy), respectively, via intrahippocampal (i.h.) injection, while the NS and EP group mice received equivalent NS injection [15]. At 24 h after the onset of seizures, under deep anesthesia with chloral hydrate (10%), mice were perfused via the ascending aorta using 4% paraformaldehyde to fix brains for the immunohistochemistry (IHC) assay (NS group, n = 5; EP group, n = 6; EP+BoxA group, n = 6; EP+BoxA group, n = 6) or decapitated to dissect out hippocampal tissues for Western blotting assay (each group, n = 6).

The present study was performed using the recombinant human disulfide HMGB1 (with a disulfide bond at positions 23 and 45 and a free thiol at position 106) provided by HMGBiotech. The purified natural protein is free from LPS (Cambrex Limulus Amoebocyte Assay QCL-1000, <0.4 ng LPS per mg protein) and contains neither nucleic acids nor interleukin-1beta (IL-1β). Previous experiments demonstrated that HMGB1 (5.5 μg per mouse) i.h. in KA-induced epileptic mice could increase the number of seizures by 70% and the total time spent in seizures by 50% [22]. In the animal experiments of this study, the same amount of HMGB1 for injection was chosen.

### 2.3 Immunohistochemistry

IHC was performed as described earlier [33]. The brain tissues were paraffin embedded and cut. After being fixed in acetone, sections of the brain regions including the cerebral cortex, hippocampus CA1/CA3/CA4, and dentate gyrus were selected for the IHC study (at least two sections per mouse). Briefly, slides were incubated with 0.03% H_2_O_2_ in PBS for 10 min at room temperature, then incubated in 5% normal goat serum in PBS for 15 min. Sections were then incubated with different primary antibodies: anti-HMGB1 (ab18256, 1:100; Abcam, HK, China), and anti-P-gp (#517310, 1:50; Calbiochem, Billerica, MA) in 3% bovine albumin serum in PBS at 4°C overnight. Immunoreactivity was detected using avidin-biotin-peroxidase technique, using 3,3ʹ-diaminobenzidine as the chromogen. Images were acquired using an upright optical microscope (DM4000; Leica Microsystems, Mannheim, Germany). For the representative section from each mouse, five nonoverlapping high-power fields (×400) from CA1 region were reviewed for cellular localization, and staining intensity of P-gp expression. For each field, the number of positively stained capillaries was counted and the percentage of P-gp positive cells was obtained. The average value of the five fields was calculated. Staining intensity was graded using a 4-point scale ：Ⅰ, ≤25% (rare); Ⅱ, 26%-50% (sparse); Ⅲ, 51%-75% (high), Ⅳ, >75% (all). Every mouse was assessed to corresponding grade according to the average percentage of P-gp positive cells.

### 2.4 Endothelial bEnd.3 cell culture and treatment with HMGB1

The bEnd.3 (Lot.no.: 60323591, ATCC^®^ CRL-2299™) mouse brain microvessel endothelial cell line [34–36] was purchased from American Type Culture Collection (Manassas, VA). Cells were incubated in Dulbecco's modified Eagle’s medium (DMEM) (Invitrogen, Carlsbad, CA) supplemented with 10% fetal bovine serum (Invitrogen), 100 U/mL penicillin, and 100 μg/mL streptomycin and maintained at 37°C in a humidified cell incubator with 5% CO_2_. Cells at passages before 10 were used for the following experiments. Recombinant human HMGB1 was applied to the cell culture medium at different concentrations (10, 100, and 500 ng/mL) for the indicated time.

### 2.5 Cell viability assay

The potential toxic effect of HMGB1 on bEnd.3 cells was analyzed using the WST-8-based cell counting kit (CCK8; Dojindo Laboratories, Kumamoto, Japan), which detects the reduction activity of dehydrogenase in viable cells [37]. Cells were seeded in 96-well plates at a concentration of 8×10^3^ cells per well. After being grown for 24 h, cells were incubated with HMGB1 (10, 100, and 500 ng/mL) or untreated control medium, respectively, for different periods of time (4, 8, 16, and 24 h). Then, 10 μL of CCK8 solution was applied to each well, followed by another 3 h’s incubation at 37°C. Optical density (OD) values were determined using a microplate reader (SpectraMax190; Molecular Devices, part of MDS Analytical Technologies, Sunnyvale, CA) using an absorbance wavelength of 450 nm.

### 2.6 Real-time quantitative polymerase chain reaction

Real-time quantitative polymerase chain reaction (qPCR) was used to detect the mRNA levels of *mdr1a* and TLR4. Total RNA was isolated from cells after indicated treatment using TRIzol reagent (Invitrogen). First-strand cDNA was synthesized using 500 ng of total RNA with PrimeScript™ RT Master Mix (TaKaRa, Otsu, Japan) in a total volume of 10 μL. The following conditions were applied for reverse transcription: 37°C for 15 min, 80°C for 5 s. The process of q-PCR was performed using a SYBR Premix Ex Taq™ kit (TaKaRa) with an ABI Prism 7300 system according to the manufacturer’s instructions. The primer sequences used were as follows: mdr1a forward, 5'-GCTGGAAGCTAACCCTTGTGA-3'; mdr1a reverse, 5'-TGCCCAAATACCAGCTGACA-3'; TLR4 forward, 5'-AGCCTTTCAGGGAATTAAGCTC-3'; TLR4 reverse, 5'-TCCCAAGATCAACCGATGGAC-3'; β-actin forward, 5'-CCTAAGGCCAACCGTGAAAAG-3'; β-actin reverse, 5'-TCTTCATGGTGCTAGGAGCCA-3'. All primers were synthesized by Sangon Biotech (Shanghai, China). The q-PCR amplification protocol consisted of 40 cycles of denaturing at 95°C for 5 s and annealing and extension at 60°C for 31 s. The relative gene expression levels were evaluated using the 2^-ΔΔT^ method [38]. The target gene expression levels were normalized to β-actin.

### 2.7 Western blotting

Western blotting was used to detect the protein levels of HMGB1, P-gp, TLR4, RAGE, phosphorus-NF-κB p65 (p-NF-κB p65), and NF-κB p65. Total proteins of hippocampal tissue and cultured bEnd.3 cells were prepared in lysis buffer (keyGEN, Nanjing, China) plus phenylmethylsulfonyl fluoride, phosphatase inhibitors, and protease inhibitors. The cytoplasmic and nuclear proteins were extracted from bEnd.3 cells with a NE-PER cytoplasmic/nuclear extraction kit from Pierce (Rockford, IL). Equal amounts of protein were fractionated by 10% (for total and cytoplasmic proteins) or 12% (for nuclear proteins) sodium dodecyl sulfate-polyacrylamide gel electrophoresis (SDS-PAGE) and transferred to polyvinylidene difluoride membranes (Millipore Co, Bedford, MA). Membranes were blocked with 5% skim milk in Tris-buffered saline containing Tween-20 (0.1%) for 2 h, and subsequently probed overnight at 4°C with primary monoclonal antibodies against P-gp (#517310, 1:50; Calbiochem), TLR4 (ab22048, 1:250; Abcam), RAGE (ab3611, 1:1000; Abcam), NF-κB p65 and phosphorus-NF-κB p65 (p-NF-κB p65) (#8242 and #3033, 1:1000; Cell Signaling Technology, Danvers, MA), HMGB1 (ab18256, 1:1000; Abcam), H3 (#4499, 1:2000; Cell Signaling Technology), and β-actin (BM0627, 1:500; BOSTER, Wuhan, China). After incubation with horseradish peroxidase-conjugated secondary antibodies for 1.5 h at room temperature, immunoblots were visualized with enhanced chemiluminescence substrate (Millipore Co) using GE ImageQuant system (LAS4000mini; GE, Fairfield, Conn). Signals from specific protein bands were quantified using ImageJ 1.47 software. Histone H3 and β-actin were utilized as loading controls for nuclear proteins and total and cytoplasmic proteins, respectively.

### 2.8 Immunofluorescence staining

The bEnd.3 endothelial cells (ECs) were seeded onto 24-well plates with cover slips. After treated with 100 ng/mL HMGB1 for 0, 30, 45, and 60 min, cells were fixed with 4% paraformaldehyde for 30 min and permeabilized with 0.5% Triton X-100 for 10 min. Later, cells were blocked with goat serum for 1 h, and incubated with primary antibody against NF-κB p65 (#8242, 1:400; Cell Signaling Technology) at 4°C overnight. After washing, the slices were further incubated with fluorescein isothiocyanate-tagged secondary antibody (AS011; ABclonal Biotech Co, Cambridge, MA) for 1 h and counterstained with 4',6-diamidino-2-phenylindole. The slices were then immediately visualized and images were captured from an upright fluorescence microscope (DM4000; Leica Microsystems).

### 2.9 Electrophoretic mobility shift assay

NF-κB activity was measured using electrophoretic mobility shift assay (EMSA) as previously described with some modifications [33]. The sequence of the double-stranded NF-κB probe was designed as follows: sense, 5′-AGT TGAGGGGACTTTCCCAGGC-3′; antisense, 3′-TCAACTCCCCTGAAAGGGTCCG-5′ (Viagene, Changzhou, China). Nuclear proteins from bEnd.3 cells were collected as described before. Extracted nuclear protein (10 μg) was incubated with 1× binding buffer, 50 ng/μL poly (dI-dC), 2.5% glycerol, 0.05% Nonidet P-40, 5 mM MgCl_2_, 0.2 mg/mL bovine serum albumin, and 400 fmol biotin-labelled NF-κB probe for 20 min at room temperature using a LightShift^®^ Chemiluminescent EMSA kit (Pierce). The protein probes reaction mixture was separated in a 6.5% nondenaturing polyacrylamide gel and transferred to a Nylon membrane (Thermo Fisher Scientific, Rockford, IL). Migration of the biotin-labeled probes was detected according to the manufacturer’s protocol. For competition experiments, nuclear extracts were incubated with 200-fold excess of unlabeled NF-κB oligonucleotide added prior to the labeled probe.

### 2.10 Plasmid construction, transfection, and luciferase reporter assay

The expression vector for NF-κB p65 (pcDNA3.1-p65) and corresponding empty vector (pcDNA3.1) was obtained from Bioworld Technology, Inc. (NM, USA). The design of reporter plasmid constructs was based on the known domains of the mouse *mdr1a* promoter and putative NF-κB binding site as shown in Table 1. Mouse genomic DNA was used as the template. The *mdr1a* promoter fragment was amplified by PCR using the following primers: forward 5’-GCGTGCTAGCCCGGGCTCGAGCAACCCTATAGGTGGAACAACA-3’, reverse 5’-CAGTACCGGAATGCCAAGCTTGCCTGGGCTTTCTGTGGACAG-3’. The *mdr1a* promoter with NF-κB p65 binding site mutant was generated with the primers: forward 5’-ATACTG**CCCTGACAGGA**CTTTCTGGTTTGCTTTGTTTTG-3’, reverse 5’- GAAAGTCCTGTCAGGGCAGTATATAAATGGAGTATTTCAG-3’. The promoter fragments were then subcloned into the KpnI/HindIII sites of PGL3-Basic vector (Promega, WI, USA) to construct reporter plasmid pGL3-mdr1a-luc (with wild-type *mdr1a* promoter) and pGL3-NFκB^mut^-mdr1a-luc (with an eleven-nucleotide mutation in the NF-κB p65 binding site). All constructs were verified by DNA-sequencing analysis.

**Table 1:** Sequence of mouse *mdr1a* (Abcb1a; GenBank accession No.: NC_000071.5) promoter.

-1000 caaccctataggtggaacaacattatgagctaaccagtaccccggagctc
-950 ttgactctagctgcatatatatcaaaagatggcctagtcggccatcactg
-900 gaaagagaggcccattggacttgcaaactttatatgccccagtacagggg
-850 aataccagggccaaaaagggggagtgggtgggcaggggagtgggggtggg
-800 tggatatgggggacttttggtatagcattggaaatgtaaatgagttaaat
-750 acctaataaaaaatggaaaaaaaaataaaataaaaataagatgaaactgg
-700 aaaaaaaaagttatgtttaataattccaattgaactgtaagaatttcaga
-650 tgccctggaaaaacatggacattggtttagtacctaaaagttcaaaatat
-600 tatatatttttaaataccattttacactgaaatactccatttatatactg
-550 ***gggactgtcct***ctttctggtttgctttgttttgtttaataaaagaaataa
NF-κB binding site
-500 accaatctacctgaggaactgtgaactatattgaagaaaagcccctgcac
-450 gggggttctcttaccttttcaagagtgcttcaaagaagggaaatttactg
-400 acaggcaaggtctgtacccattgtttaattgtctgttagatgttatgcat
-350 agaatacgtcttttaacttagccaaatgcagaaggccaagtgcatatcta
-300 caaacacataactctatatatagacatgtgcatggccgtgtagagatgag
-250 actcttgcaagtgtgtctctaatgattcggggatatgagtttgtctaatt
-200 gacctttgagaggggaaaccagactgcacatttcatctacaaatccaacc
-150 tgtttcgcaatttctccagcaataatacttgagtcaagctgggccgggag
-100 ctggttaacctccaggtcaaactcactggctgggcgggactgcgcctggg
-50 cgtagattgagcatgctaaatttactctcctgtccacagaaagcccaggc
+1 ***a***cagtggaacagcggtttccaggagctgctggtcccatcttccaaggctc
transcription start site
+51 tgctcaactcagagccgcttcttccaaagtctacatcttggtggactttg
+101 cagaggaaaccgggaggtagagacacgtgag

The transcriptional initiation site is designated by the number +1.The fragment spanning −1000 to -1 was fused to the luciferase gene in the pGL3-Basic vector (forming pGL3-mdr1a-luc). Putative NF-κB binding site is located at -550∼-540.

For transfection, bEnd.3 cells were seeded at 3×10^4^/well in a 24-well plate. Co-transfections were carried out with 400 ng/well of reporter plasmids and 200 ng/well of NF-κB p65 expression vector or the empty vector using Lipofectamine™ 2000 (Invitrogen). The cells were harvested 72 h after transfection, and luciferase activity of transient transfectants was measured using Dual-Luciferase^®^ Reporter Assay System (Promega) according to the luciferase assay protocol. To standardize the transfection efficiency, 8 ng/well of phRL-TK (Renilla luciferase) plasmid was co-transfected into the cells as an internal control. The luminescent intensity of firefly luciferase was normalized as a ratio to that of Renilla luciferase [39, 40].

### 2.11 Statistical analysis

Quantitative data were expressed as mean±standard deviation from at least three independent experiments. Measurement data were analyzed using one-way analysis of variance followed by Fisher’s protected least significant difference test (for data with equal variances) or Welch followed by Dunnett’s T3 (for data that did not have equal variances) or independent samples t test; Ranked data were analyzed using Kruskal-Wallis test and Wilcoxon rank sum test. Analysis was performed with SPSS 13.0 (SPSS, Chicago, IL). Differences were considered statistically significant when *P*<0.05.

## Results

### 3.1 Expression and distribution of HMGB1 in the KA-induced mouse epileptic brain tissues

**Fig 1:**
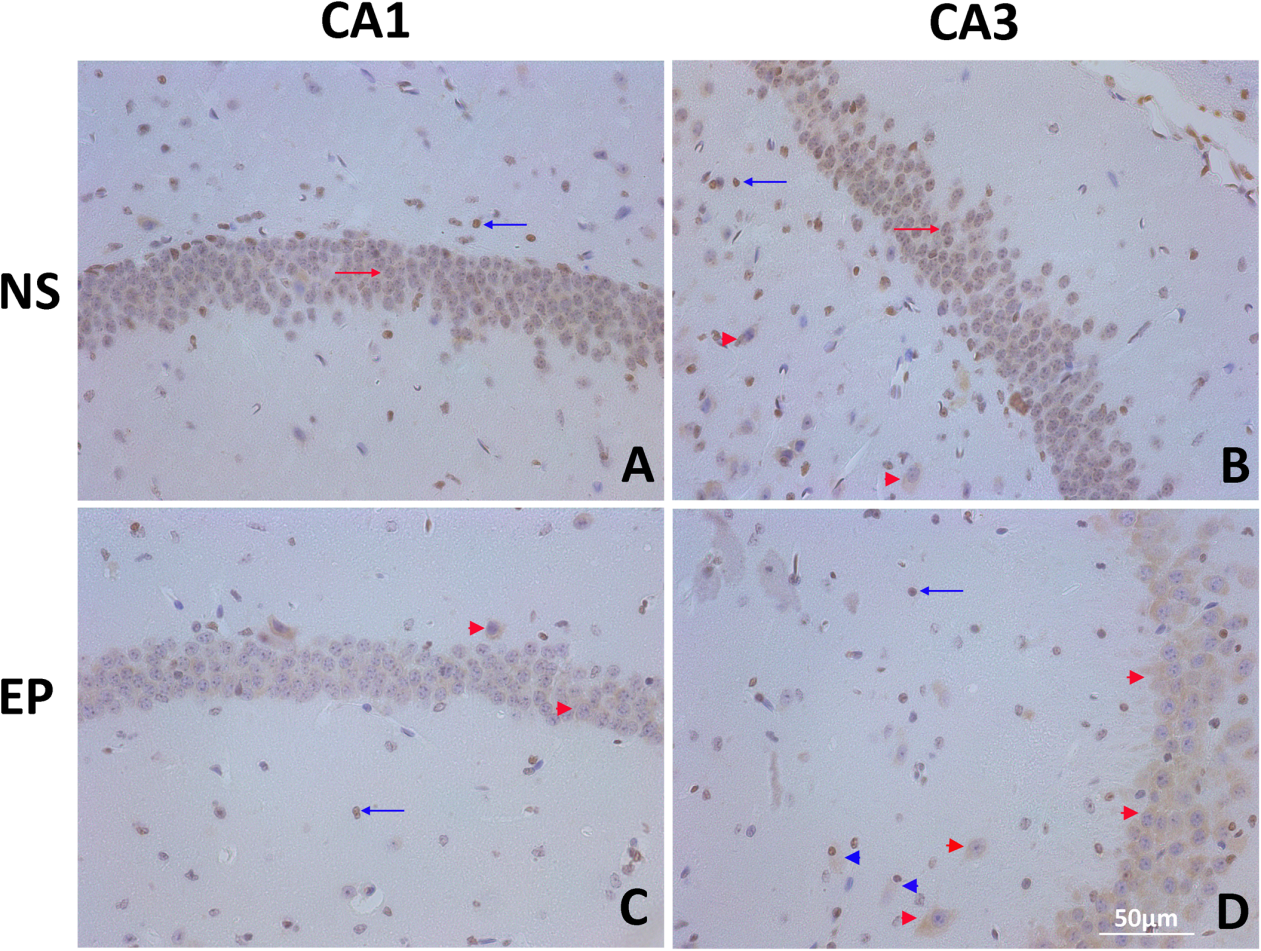
Expression and distribution of HMGB1 in the KA-induced mouse epileptic brain tissues. (A&B) Immunohistochemical study detecting HMGB1 in the CA1 (A) and CA3 (B) hippocampal regions from the NS group mice (*n* = 5). HMGB1 immunoreactivity was detected mostly in the nuclei of the pyramidal neurons. (C&D) Immunohistochemical study detecting HMGB1 in the CA1 (C) and CA3 (D) hippocampal regions from the EP group mice (*n* = 6). Cytoplasmic staining of HMGB1 was substantially increased in the pyramidal neurons compared with the NS group. Glial cells with both nuclear and cytoplasmic staining were also found. Scale bar: 50 μm. Red arrows: neurons with nuclear staining; Blue arrows: glial cells with nuclear staining; Red arrowheads: neurons with both nuclear and cytoplasmic staining; Blue arrowheads: glial cells with both nuclear and cytoplasmic staining. EP, KA-induced epileptic seizure group; HMGB1, high-mobility group box-1; KA, kainic acid; NS, normal saline control group.

To investigate the expression level and changes in the distribution pattern of HMGB1 in post-seizure mouse brain, KA-induced epileptic seizure model was used. The distribution of HMGB1 in the CA1 and CA3 regions from NS and EP group mice was observed using IHC. In the NS group, HMGB1 immunoreactivity was detected mostly in the nuclei of the pyramidal neurons. In the stratum radiatum (sr), stratum lacunosum (sl), and stratum moleculare (sm), decentralized glial cells with nuclear staining as well as neurons with both nuclear and cytoplasmic staining were observed; HMGB1-negative cells were also observed (Fig 1A and 1B). In the EP group, cytoplasmic staining of HMGB1 was substantially increased in the pyramidal neurons (Fig 1C and 1D). Besides of glial cells with nuclear staining, glial cells with both nuclear and cytoplasmic staining in sr, sl, and sm (Fig 1D) of CA3 regions were also found. These results indicated the nuclear to cytoplasmic translocation of HMGB1 in neurons and glial cells, and predicted its subsequent release into the extracellular space.

### 3.2 Pre-treatment with HMGB1 enhanced KA-induced up-regulation of P-gp in the epileptic brain

**Fig 2:**
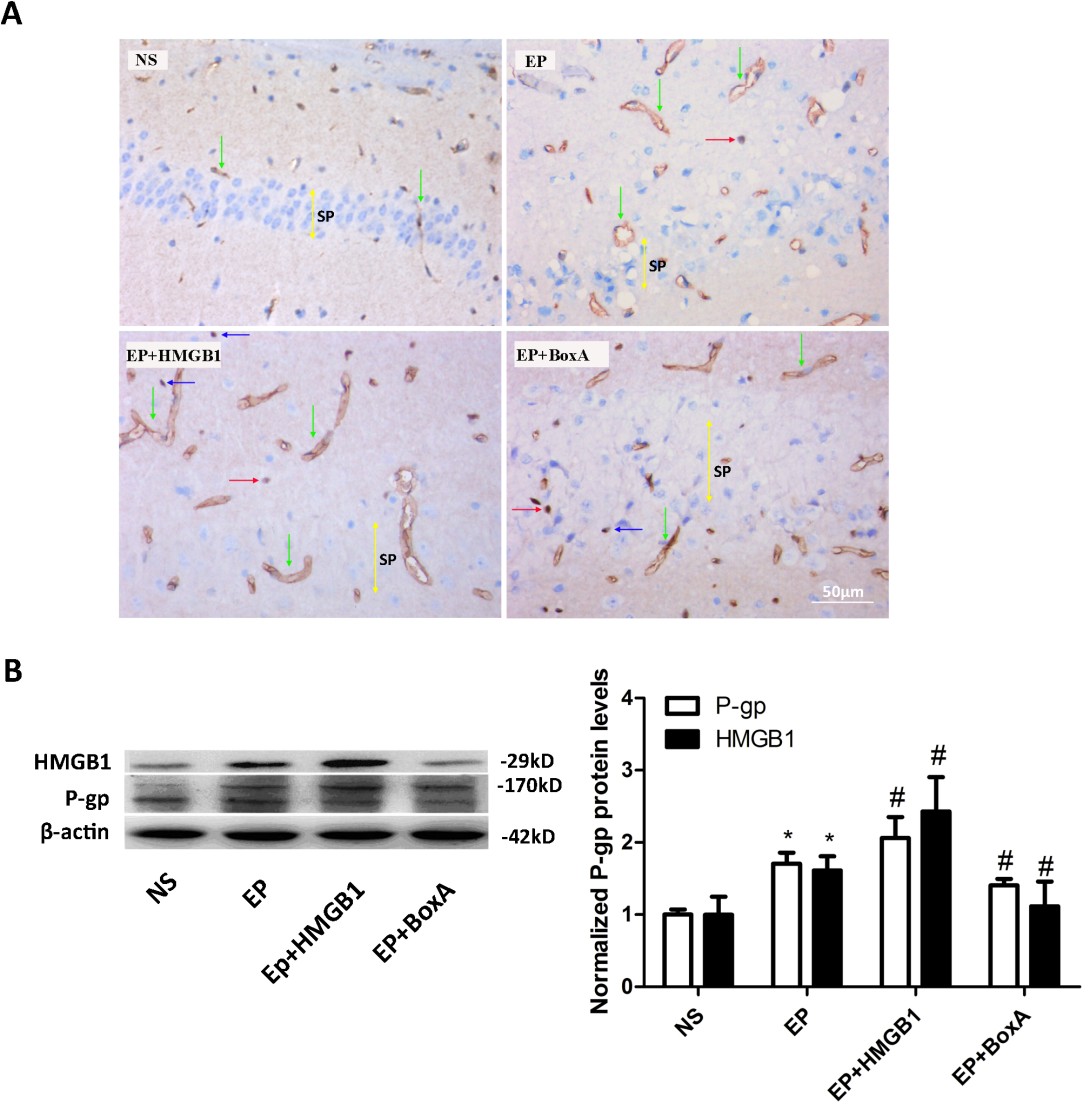
Pre-treatment with HMGB1 enhanced KA-induced up-regulation of P-gp in the epileptic brain. (A) Immunohistochemical staining of P-gp in CA1 hippocampal areas of each group mice (NS group, *n* = 5; EP group, *n* = 6; EP+BoxA group, *n* = 6; EP+BoxA group, *n* = 6). HMGB1 injection enhanced the over-expression of P-gp in the EP group mice while BoxA attenuated it. Scale bar: 50 μm. NS: normal saline control group; EP: KA-induced epileptic seizure group; EP+HMGB1: EP group pretreated with HMGB1; EP+BoxA: EP group pretreated with BoxA. Green arrows: vascular endothelial cells; Red arrows: neurons; Blue arrows: glial cells; Yellow bidirectional arrows: Stratum pyramidale (sp). (B) Western blotting detects the protein levels of HMGB1 and P-gp in the mice brains of each group 24 h after seizure onset (B left panel, *n* = 6). Protein levels were quantified and normalized to that in the NS group (B right panel). Injection of HMGB1 and BoxA increased and decreased the protein levels of HMGB1 and P-gp in the EP group mice respectively. *P<0.05 vs. NS group; ^#^ *P*<0.05 vs. EP group. HMGB1, high-mobility group box-1; KA, kainic acid; P-gp, P-glycoprotein.

As detected by IHC, P-gp is mostly expressed in vascular endothelial cells in the hippocampal regions. Very few neurons and glial cells with P-gp staining were observed. In the NS group, only several P-gp immunopositive capillaries were detected in the CA1 region. Compared to the NS group, the numbers of P-gp-positive capillaries significantly increased in the EP group. This KA-induced P-gp up-regulation was further increased by the HMGB1 injection in the HMGB1+KA group and was abolished by the pre-treatment of BoxA (an antagonist to HMGB1) in the BoxA+KA group (Fig 2A). Result of the semi-quantitative assay for the IHC of P-gp expression was summarized in Table 2: EP group has more mice with stronger P-gp staining (been graded to the grade III and grade IV) as compared to the control NS group; the EP+HMGB1 group has even more mice been assessed in the grade III and grade IV, while the EP+BoxA group has less. Consistent to the above IHC findings, Western blotting detecting brain P-gp levels also showed an elevation of P-gp level in the EP group as compared to the NS group, and this up-regulation was enhanced by the pre-treatment of HMGB1 and was abolished by the BoxA pre-treatment (*P*<0.05, Fig 2B). Similarly, the brain HMGB1 level also increased in the EP group as compared to the NS group. This level further increased in the EP+HMGB1 group and attenuated in the EP+BoxA group. Collectively, these data show that HMGB1 is closely related to the level of expression of P-gp in the KA-induced epileptic brain.

**Table 2:** The staining intensity of P-gp in the CA1 regions from mice in different groups.

Group	P-gp			
	Ⅰ	Ⅱ	Ⅲ	Ⅳ
NS(*n* = 5)	3	2	0	0
EP*(*n* = 6)	0	3	2	1
EP+HMGB1^#^ (*n* = 6)	0	1	3	2
EP+BoxA^#^ (*n* = 6)	0	4	2	0

The number of mouse in different staining grade was shown.

*P<0.05 vs. NS group

^#^ *P*<0.05 vs. EP group.

### 3.3 HMGB1 had no significant effect on the viability of bEnd.3 cells

**Fig 3:**
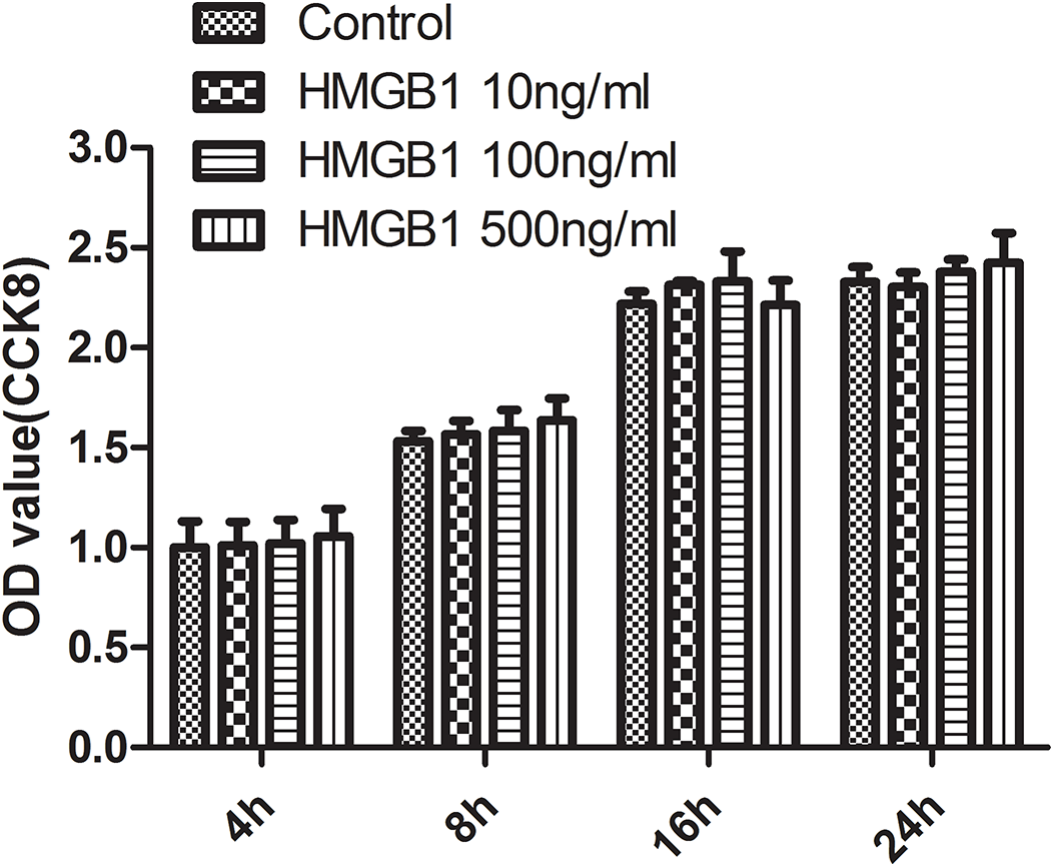
HMGB1 had no significant effect on the viability of bEnd.3 cells. Cells were treated with different concentrations of HMGB1 for the indicated time periods. Cell viability was determined using CCK8 assay. Data from three independent experiments was shown as mean±SD; *n* = 3. CCK8, cell counting kit; HMGB1, high-mobility group box-1; SD, standard deviation.

As shown in Fig 3, the incubation with HMGB1 does not decrease the viability of bEnd.3 cells significantly at any tested concentrations and time points, as compared to the control cells. The concentration of extracellular HMGB1 in epileptic brain remains unknown, but its local concentration is expected to be substantial, since a significant proportion of nuclear HMGB1 is released or secreted into a very limited extracellular space between the stressed adjoining cells. Andersson et al. [41] have found that concentrations of HMGB1 as low as 10 ng/mL significantly stimulated the release of TNF in monocytes, and 1 μg/mL of HMGB1 significantly activated the synthesis of many cytokines such as TNF, IL-1α, IL-1β, IL-1RA, IL-6, and IL-8. Yang et al. [42] have reported that HMGB1 ranging from 50 to 1000 ng/mL could markedly increase the adhesion of polymorphonuclear leukocytes to ECs from thoracic aorta and the expression of intercellular adhesion molecule-1 and E-selectin in ECs. Therefore, the dose range of HMGB1 from 10 to 500 ng/mL was kept in the following *in vitro* experiments.

### 3.4 HMGB1 upregulated the expression of P-gp, TLR4, and RAGE in bEnd.3 cells

**Fig 4:**
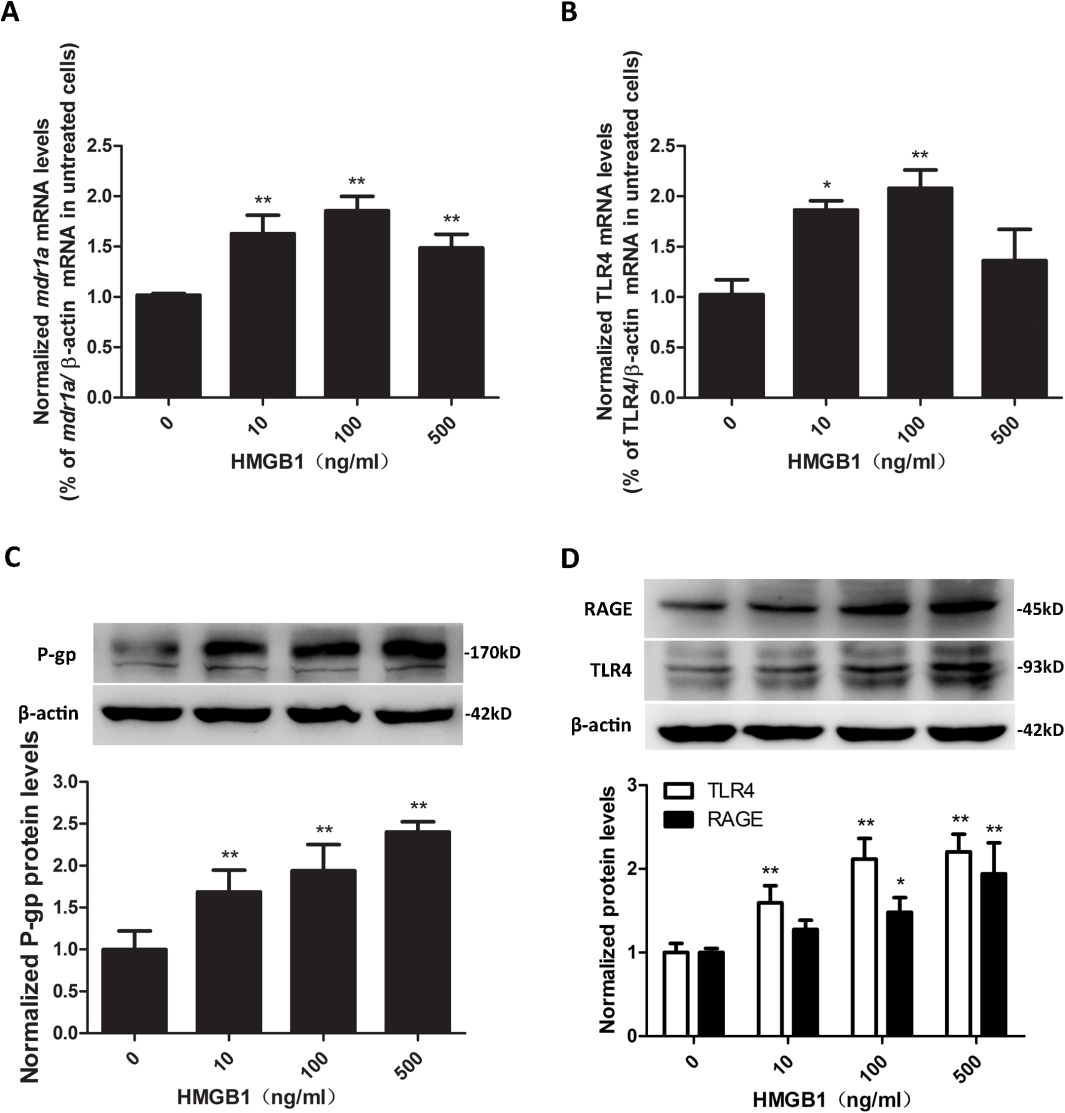
HMGB1 upregulated the expression of P-gp, TLR4, and RAGE in bEnd.3 cells. (A&B) The mRNA levels of *mdr1a* (A) and TLR4 (B) in bEnd.3 cells treated with different concentrations of HMGB1 were measured using q-PCR and normalized to the cells treated without HMGB1. HMGB1 at all tested concentrations (from 10 to 500 ng/mL) increased *mdr1a* mRNA and TLR4 mRNA levels in bEnd.3 cells as compared to cells treated without HMGB1. (C&D) The protein levels of P-gp (C), TLR4, and RAGE (D) in bEnd.3 cells treated with different concentrations of HMGB1 were analyzed using Western blotting with β-actin as a loading control. Protein levels were quantified and normalized to the cells treated without HMGB1. Treatment with HMGB1 at all tested concentrations increased P-gp, TLR4, and RAGE protein levels in bEnd.3 cells. Data were shown as mean±SD; *n* = 3. *P<0.05, **P<0.01 vs. cells treated with medium only. HMGB1, high-mobility group box-1; *mdr1a*, *multidrug resistance1a*; P-gp, P-glycoprotein; SD, standard deviation; TLR4, toll-like receptor 4; RAGE, receptor for advanced glycation end products.

To examine the effects of HMGB1 on P-gp, TLR4, and RAGE expression levels, bEnd.3 cells were treated with 10, 100, and 500 ng/mL HMGB1 for 4h (for detection of mRNA levels) and 16 h (for detection of protein levels), and *mdr1a* mRNA, TLR4 mRNA and P-gp, TLR4, and RAGE protein levels were detected using q-PCR and Western blotting, respectively. As shown in Fig 4A, treatment with HMGB1 at all tested concentrations significantly increased *mdr1a* mRNA levels in bEnd.3 cells as compared to cells treated with normal media (*P*<0.01, Fig 4A). TLR4 mRNA levels in cells treated with 10, 100 ng/mL HMGB1 were also significantly higher than that in cells treated without HMGB1 (*P*<0.05, Fig 4B). Interestingly, the maximal upregulating effect of HMGB1 on *mdr1a* and TLR4 mRNA levels was observed at the concentration of 100 ng/mL but not 500 ng/mL. However, HMGB1 increased P-gp TLR4, and RAGE protein levels in bEnd.3 cells in a concentration-related manner as detected using Western blotting (*P*<0.05, Fig 4C and 4D). The inconsistency of mRNA and protein expression changes of P-gp and TLR4 in the 500 ng/mL HMGB1 group may be related to the selected specific examination time point (the peak of mRNA level in the 500 ng/mL HMGB1 group may not occur at 4 h), or cell feedback inhibition.

### 3.5 HMGB1 enhanced the activation of NF-κB in bEnd.3 cells

**Fig 5:**
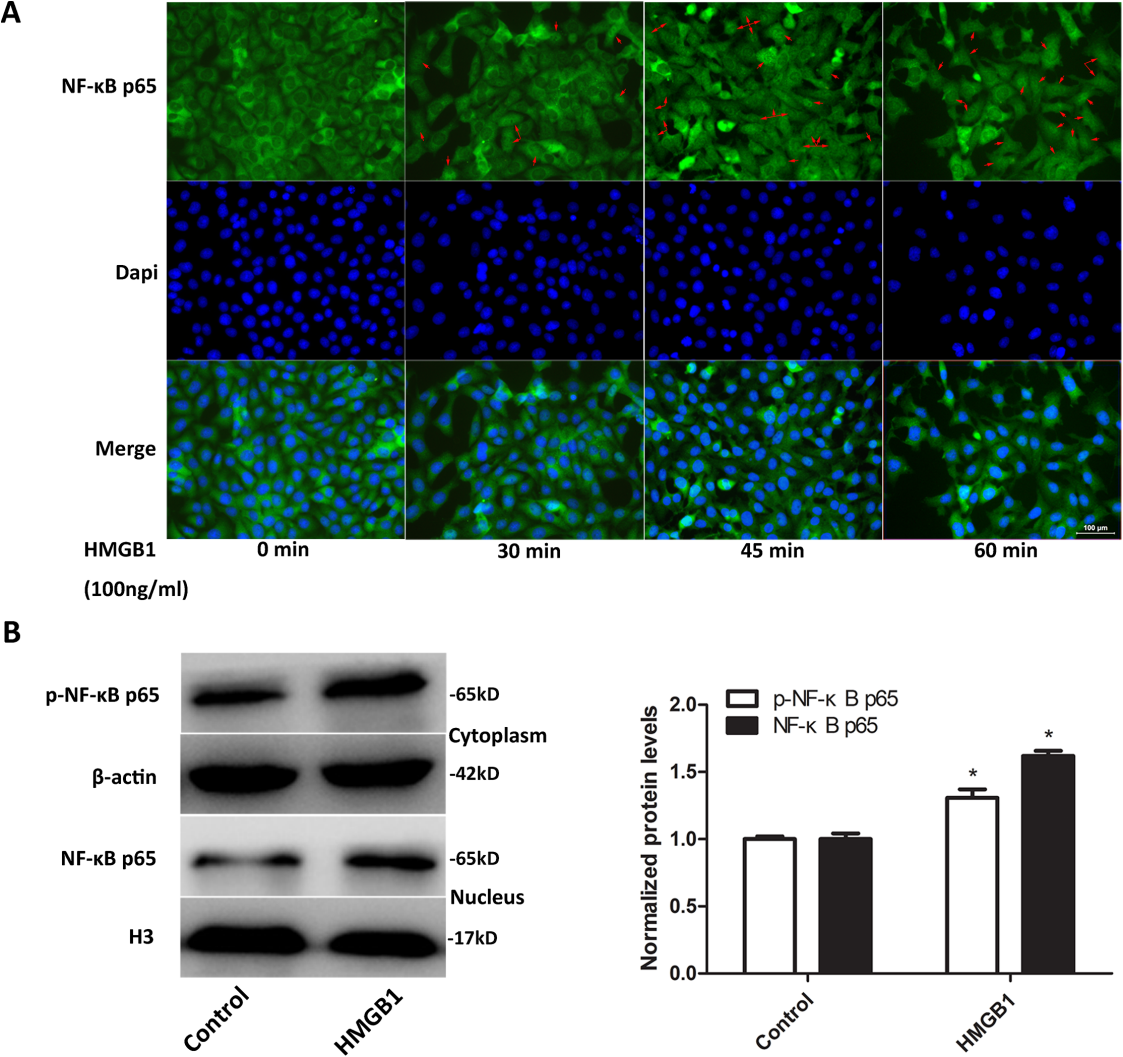
HMGB1 enhanced phosphorylation and nuclear translocation of NF-κB p65 in bEnd.3 cells. (A) NF-κB p65 subcellular distribution in bEnd.3 cells treated with HMGB1 for indicated durations was observed using immunofluorescence staining. HMGB1 promoted cytoplasmic to nuclear translocation of NF-κB p65 in bEnd.3 cells. Representative pictures are shown. Scale bar: 100 μm. Red arrows: cells with nuclear NF-κB p65 staining. (B) Protein levels of phosphor-NF-κB p65 and NF-κB p65 in the cytoplasm and nuclear fractions from bEnd.3 cells treated with HMGB1 for 60 min. H3 and β-actin were utilized as loading controls for nuclear and cytoplasmic proteins, respectively. Protein levels were normalized to the cells treated without HMGB1. HMGB1 increased cytoplasmic p-NF-κB p65 and nuclear NF-κB p65 levels in bEnd.3 cells. Data were shown as mean±SD; *n* = 3. *P<0.05 vs. cells treated with medium only. HMGB1, high-mobility group box-1; NF-κB, nuclear factor-kappa B; SD, standard deviation.

To identify the effect of HMGB1 on the activation of NF-κB, translocations to nuclei of NF-κB p65 in bEnd.3 cells that underwent 100 ng/mL HMGB1 stimulation for 0, 30, 45, and 60 min were observed using immunofluorescence method. Cells treated with 100 ng/mL HMGB1 for 60 min were harvested for the cytoplasmic and nuclear protein extraction and detection of p-NF-κB p65 and NF-κB p65 protein levels using Western blotting. As shown in Fig 5A, the expression of NF-κB p65 was only located in the cytoplasm in unchallenged bEnd.3 cells. After exposure to HMGB1, NF-κB p65 signal was observed also in the nuclei. The number of cells with nuclear NF-κB p65 signal increased gradually according to the extension of time of incubation with HMGB1. At 60 min after HMGB1 treatment, the translocation of NF-κB p65 was already quite obvious. In addition, results of Western blotting further confirmed that p-NF-κB p65 in the cytoplasm and NF-κB p65 in the nuclei fraction were increased in HMGB1-treated cells compared with the control (*P*<0.05, Fig 5B). These data suggested that the transcription factor NF-κB was activated in HMGB1-treated bEnd.3 cells.

### 3.6 Pre-treatment with LPS-RS, FPS-ZM1, or SN50 attenuated HMGB1-induced up-regulation of P-gp in bEnd.3 cells

**Fig 6:**
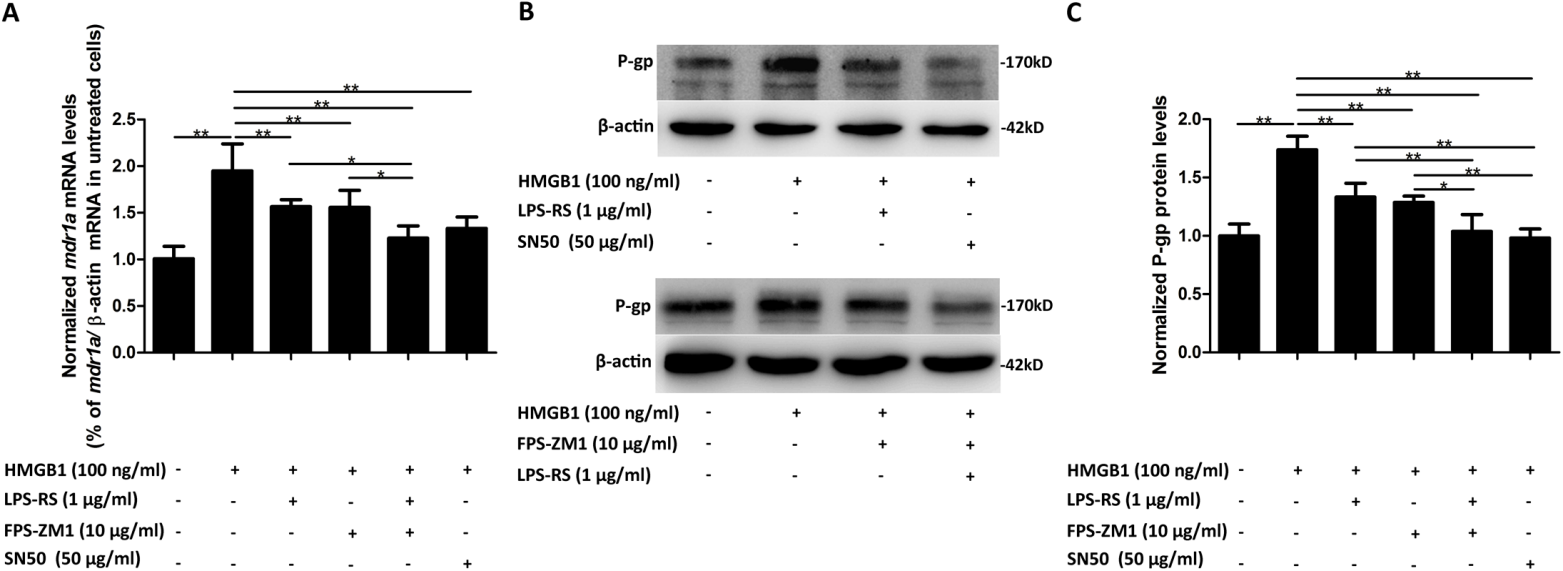
Pre-treatment with LPS-RS, FPS-ZM1, or SN50 attenuated HMGB1-induced up-regulation of P-gp in bEnd.3 cells. (A) The mRNA levels of *mdr1a* in bEnd.3 cells treated with HMGB1 plus different inhibitors. Inhibition of TLR4, RAGE, and NF-κB attenuated HMGB1-induced up-regulation of *mdr1a* mRNA in bEnd.3 cells. LPS-RS: TLR4 antagonist; FPS-ZM1: RAGE inhibitor; SN50: NF-κB nuclear translocation inhibitor. (B&C) The protein levels of P-gp in bEnd.3 cells treated with HMGB1 plus different inhibitors. Representative examples are shown (B) and protein levels were normalized to the cells treated without HMGB1 (C). Inhibition of TLR4, RAGE, and NF-κB attenuated HMGB1-induced up-regulation of P-gp protein in bEnd.3 cells. Data were shown as mean±SD; *n* = 3. *P<0.05, **P<0.01. HMGB1, high-mobility group box-1; LPS-RS, lipopolysaccharide from *Rhodobacter sphaeroides*; *mdr1a*, *multidrug resistance1a*; NF-κB, nuclear factor-kappa B; P-gp, P-glycoprotein; SD, standard deviation; TLR4, toll-like receptor 4; RAGE, receptor for advanced glycation end products.

To confirm the involvement of TLR4, RAGE, and NF-κB in HMGB1-induced up-regulation of P-gp, bEnd.3 cells were pretreated with either TLR4-specific antagonist LPS-RS (1 μg/mL, which is thought to form inactive complexes with TLR4/MD2), FPS-ZM1 (10 μg/mL, a high-affinity RAGE-specific inhibitor) or a joint treatment of LP-RS plus FPS-ZM1, or NF-κB nuclear translocation inhibitor SN50 (50 μg/mL) for 4 h before the incubation of HMGB1 (100 ng/mL). P-gp mRNA and protein levels were then observed. As shown in Fig 6A, the HMGB1-induced up-regulation of P-gp mRNA level in bEnd.3 cells was partially but significantly reduced by the pre-treatment with LPS-RS or FPS-ZM1 respectively (*P*<0.01), and abolished by the pre-treatment with SN50 or a combination treatment of both LPS-RS and FPS-ZM1 (*P*<0.01, Fig 6A). The similar inhibiting effect of these inhibitors were also observed on the HMGB1-induced up-regulation of P-gp protein level (*P*<0.01, Fig 6B and 6C). Results indicated that both the two receptors (TLR4 and RAGE) and the transcription factor NF-κB play important roles in HMGB1-induced overexpression of P-gp.

### 3.7 TLR4 and RAGE were involved in HMGB1-induced activation of NF-κB in bEnd.3 cells

**Fig 7:**
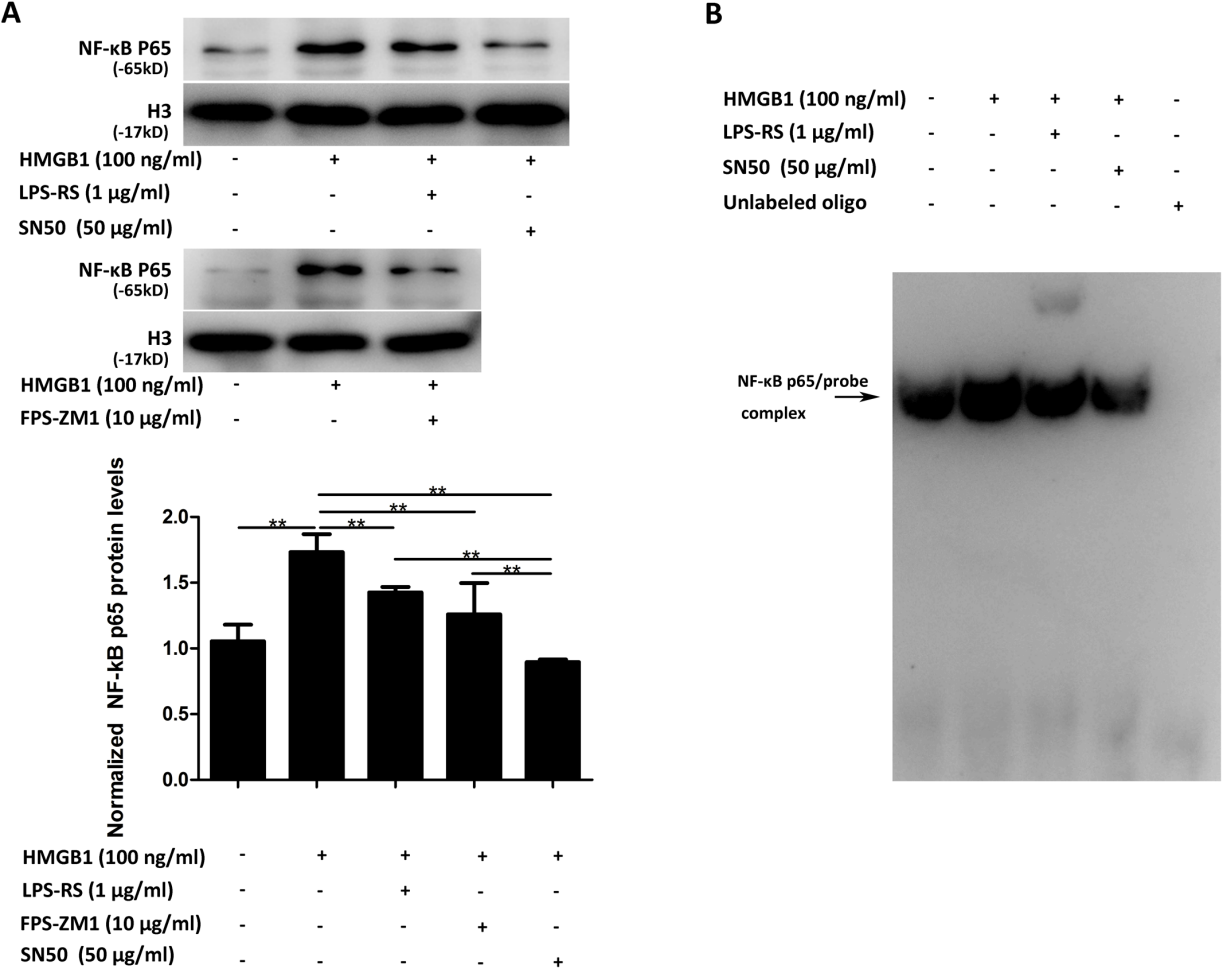
TLR4 and RAGE were involved in HMGB1-induced activation of NF-κB in bEnd.3 cells. (A) Nuclear NF-κB p65 protein levels in bEnd.3 cells treated with HMGB1 plus different inhibitors. Inhibition of TLR4 and RAGE resulted in a significant decrease in HMGB1-induced NF-κB p65 translocation to nuclei. (B) NF-κB DNA-binding activity in bEnd.3 cells treated with HMGB1 plus different inhibitors detected by EMSA. HMGB1 increased NF-κB p65 binding activity to DNA in bEnd.3 cells while inhibition of TLR4 or SN50 attenuated this effect of HMGB1. Data were shown as mean±SD; *n* = 3. **P<0.01. HMGB1, high-mobility group box-1; EMSA, electrophoretic mobility shift assay; LPS-RS, lipopolysaccharide from *Rhodobacter sphaeroides*; NF-κB, nuclear factor-kappa B; SD, standard deviation; TLR4, toll-like receptor 4; RAGE, receptor for advanced glycation end products.

Both genetic and biochemical data support that TLR and RAGE signaling pathway could eventually lead to the activation of NF-κB. In the present study, to further validate the relationships between activation of NF-κB and activation of TLR4 or RAGE, bEnd.3 cells were pretreated with LPS-RS (1 μg/mL), FPS-ZM1 (10 μg/mL) or SN50 (50 μg/mL, as a positive control) for 4 h before being incubated with HMGB1 (100 ng/mL) for another 1 h. Nuclear NF-κB p65 levels and NF-κB p65 DNA-binding activity were detected using Western blotting and EMSA assay. Western blotting showed that HMGB1-induced NF-κB p65 translocation to nuclei was significantly reduced by the treatment of LPS-RS or FPS-ZM1 (*P*<0.01, Fig 7A), while SN50 nearly totally inhibited the translocation (*P*<0.01, Fig 7A). As indicated by the EMSA assay (Fig 7B), a significant increase in NF-κB p65 binding activity to DNA was observed upon stimulation with HMGB1, and this HMGB1-induced NF-κB activation was significantly attenuated by the pre-treatment of LPS-RS or SN50.

### 3.8 Exogenous expression of NF-κB p65 activated the *mdr1a* promoter in bEnd.3 cells and the predictive NF-κB binding site was essentially involved in this process.

**Fig 8:**
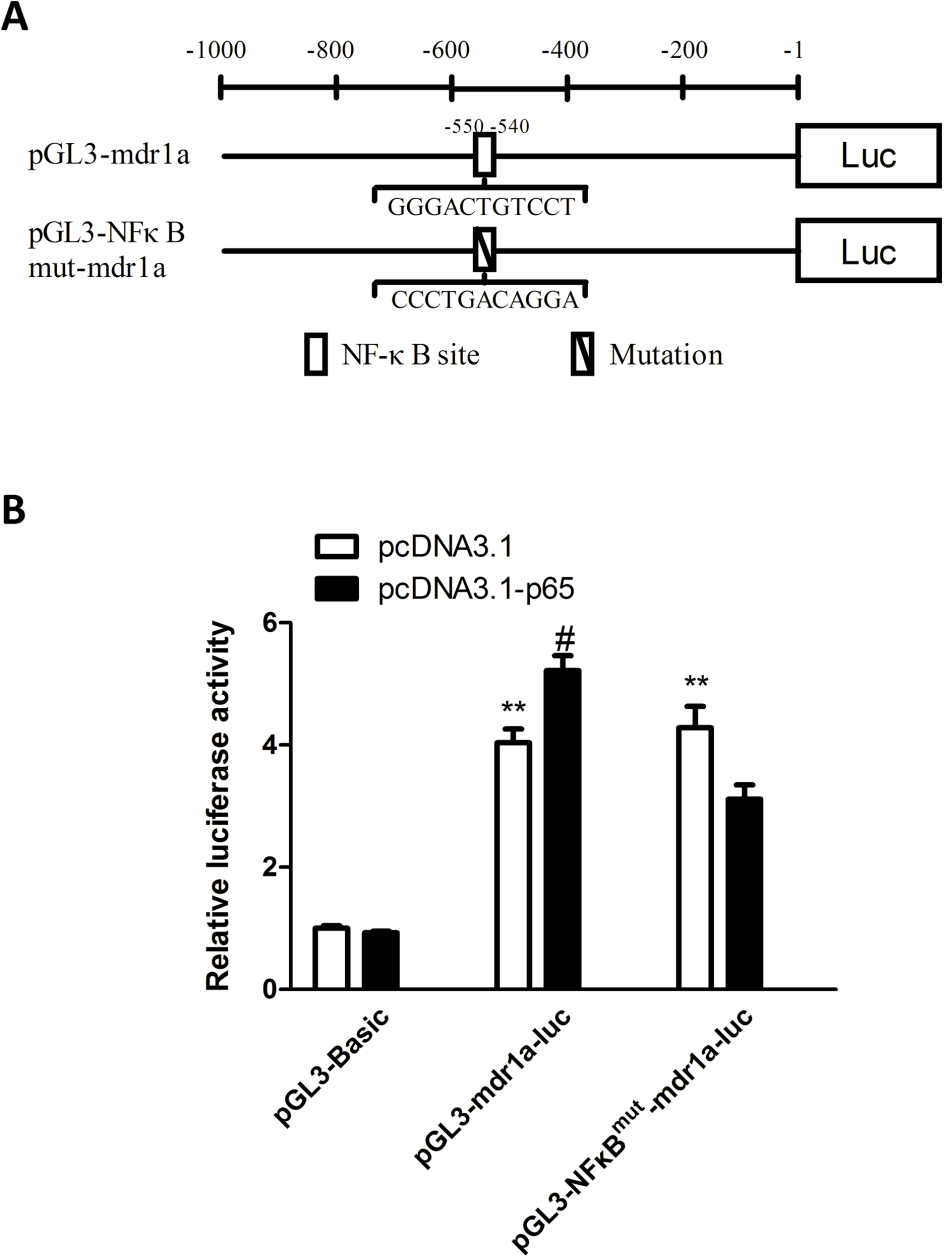
Exogenous expression of NF-κB p65 activated the *mdr1a* promoter in bEnd.3 cells and the predictive NF-κB binding site was essentially involved in this process. (A) Promoter constructs used in the study. -1 represents the first nucleotide upstream the transcription start site. Plasmid pGL3-mdr1a-luc included wild-type *mdr1a* promoter fragment and pGL3-NFκB^mut^-mdr1a-luc included an eleven-nucleotide mutation in the putative NF-κB binding site in *mdr1a* promoter. (B) Luciferase activity in transfected bEnd.3 cells was measured 72 h after the cotransfection of reporter plasmids and p65 expression vector. Overexpression of p65 in bEnd.3 cells led to a significant increase in *mdr1a* promoter activity as compared to its counterpart control. Mutation of the potential NF-κB binding site in *mdr1a* promoter abolished the promotor activation. Data were shown as mean±SD; *n* = 3. **P<0.01, compared with promotorless pGL3.0-Basic vector; ^#^ *P*<0.05, compared with pcDNA3.1 negative control. NF-κB, nuclear factor-kappa B.

Co-transfection of NF-κB p65 expression plasmid with reporter plasmids (pGL3-Basic, pGL3-mdr1a-luc or pGL3-NFκB^mut^-mdr1a-luc) was utilized to monitor the effect of p65 on the activity of *mdr1a* promoter and identify the interacting site. As shown in Fig 8B, pGL3-mdr1a-luc and pGL3-NFκB^mut^-mdr1a-luc had significantly higher basal luciferase activity than the promotorless pGL3.0-Basic vector (*P*<0.01, Fig 8B). The overexpression of NF-κB p65 increased *mdr1a* promotor activity when co-transfected with pGL3-mdr1a-luc, compared with the empty vector control (*P*<0.05, Fig 8B). Mutation of the putative NF-κB binding site abolished the up-regulation of luciferase intensity induced by NF-κB p65 (Fig 8B). These findings demonstrated that NF-κB p65 directly enhances the activity of *mdr1a* promoter through the predictive NF-κB binding site.

## Discussion

In the present study, the experiments were started by comparing the expression levels and distribution patterns of HMGB1 in the hippocampal tissues between the NS control mice and KA-induced epileptic mice. HMGB1 was found to be significantly upregulated in the post-seizure brain. Increased staining of perinuclear HMGB1 in neurons and emerged glial cells with both nuclear and perinuclear HMGB1 staining implied the nuclear-to-cytoplasmic translocation of HMGB1. Knowledge about the expression of HMGB1 in epilepsy is still highly limited. These results demonstrated that up-regulation and release of HMGB1 exist in the post-seizure mouse hippocampal tissues, being consistent with Maroso’s study [15].

To examine the relationship between HMGB1 and overexpression of P-gp in post-seizure brain tissues, either intracephalic extracellular HMGB1 was increased by i.h. injection of recombinant HMGB1 or endogenous HMGB1 activity was inhibited by the injection of BoxA [41], preceding the induction of KA. Here we reported that, 24 h after the onset of KA-induced seizure, the expression of P-gp in the hippocampus area was significantly increased, and this up-regulation of P-gp was enhanced by the pre-treatment with HMGB1 and attenuated by the pre-treatment with BoxA, suggesting that HMGB1 plays an important role in the upregulated expression of P-gp in the post-seizure brain. Interestingly, it has been reported that HMGB1 could directly increase the gene transcription and expression of P-gp in tumor cells [11]. Whether HMGB1 signaling could directly upregulate P-gp expression in neuronal cells is still unclear and worthy further investigation.

Previous researches substantiated that seizures may induce overexpression of P-gp in four different cell types, i.e., EC, perivascular astrocytes, parenchymal astrocytes, and neurons [3, 17, 43, 44]. However, in the IHC assay of this study, P-gp is mainly observed in vessels in epileptic brain tissues while astrocytic expression was invisible. This phenomenon may be related to the acetone fixation method used in the experiment, as Volk et al. have demonstrated that various fixation conditions and staining variables have a striking effect on the immunohistochemical localization of P-gp in rat brain [3]. And yet for all that, it is acknowledged that overexpression of P-gp in endothelial cells is the first line of defense for inhibiting the penetration of AEDS to parenchyma and contributes much to drug-resistance. Thus, the mouse brain microvascular endothelial cell line, bEnd.3, was used for the subsequent *in vitro* experiments to investigate the direct effect of HMGB1 on the expression of P-gp in vascular endothelial cells and to explore the signaling pathway involved. Omidi et al. [35] have demonstrated that this cell line possesses a range of relevant transporter systems (including P-gp) significant to the drug penetration of central nervous system and thus can be used as an appropriate cell model for expression modulation studies regarding selected transporters. In the present experiment, HMGB1 sitimulation significantly increased the expression of P-gp in bEnd.3 cells, and the effect was concentration-related. These data are similar to a recent study that showed the upregulating effect of extracellular HMGB1 on the expression levels of P-gp in gastric adenocarcinoma cells [11]. During seizures, in addition to the release of HMGB1 from excitotoxic neuronal cell death and induction of overexcitation, subsequently increased expression and secretion of HMGB1 by glial cells is expected to support a second and stable wave of extracellular HMGB1 [15]. Previous studies mostly focused on the pro-inflammatory and pro-convulsant effects of HMGB1 in the pathological process of epilepsy, while the present study firstly demonstrates the important role of HMGB1 in seizure-induced overexpression of P-gp.

**Fig 9:**
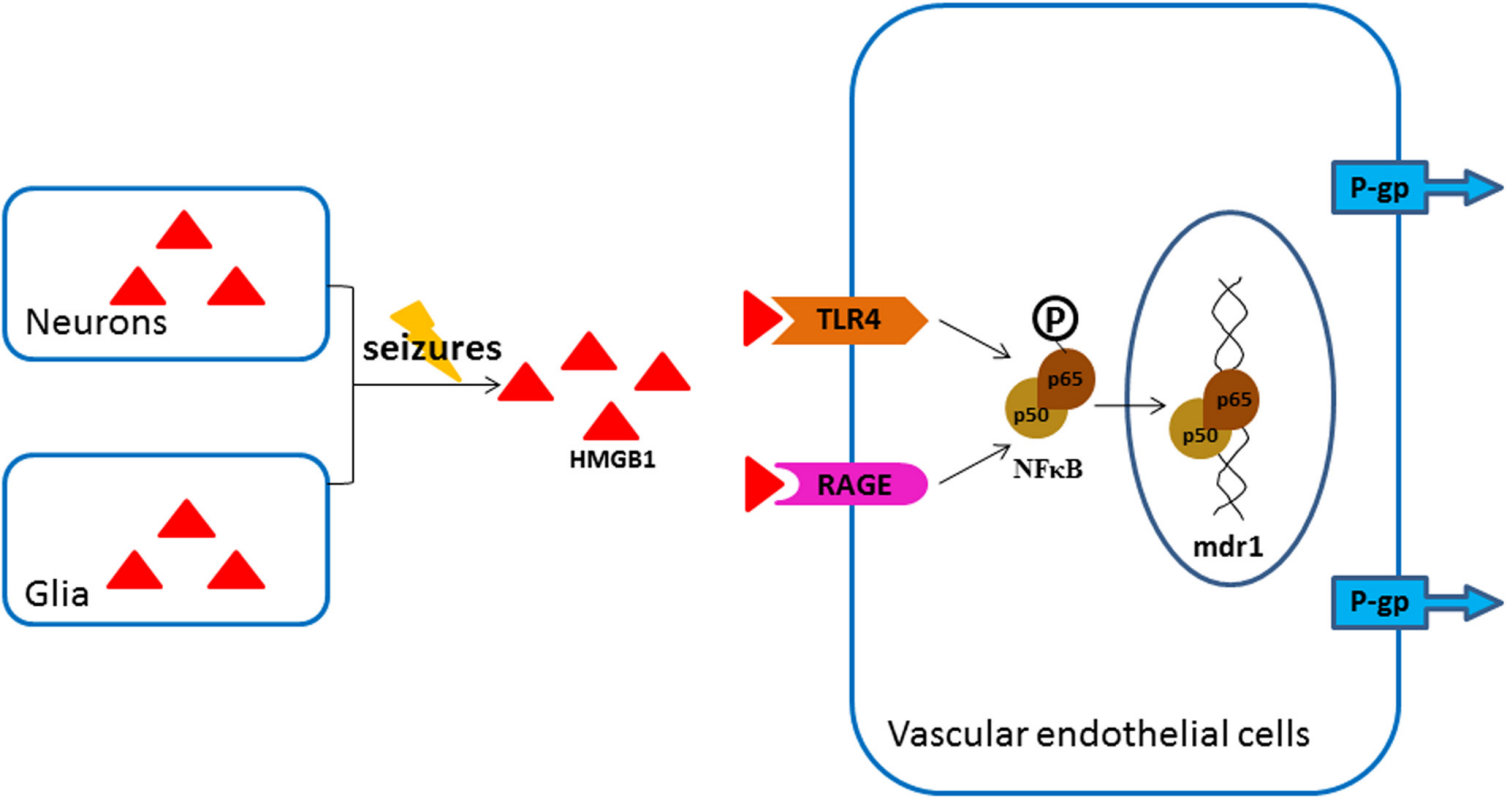
A proposed model of P-gp up-regulation by HMGB1 in epileptic brain.

Many studies have reported that HMGB1 exerts its effects through various receptors, among which, RAGE and TLR4 may be the most representative ones. Upon activation, TLR4 initiates either MyD88 or TRIF dependent pathways and leads to the activation of transcription factor NF-κB, AP-1, or IRF3 [20]. The TLR4/NF-κB signaling pathway has been shown to mediate regulation of P-gp in renal proximal tubule epithelial cells [45]. RAGE engagement by ligands activates various signaling pathways, and NF-κB is the first signal transduction molecule found to be activated. The stimulation of RAGE is able to activate the mitogen-activated protein kinase signaling cascades which converge in IκB kinase complex to inhibit IκB, thereby release and activate NF-κB [21]. The present study supports the critical roles of TLR4, RAGE and NF-κB in mediating the upregulating effect of HMGB1 on P-gp in endothelial bEnd.3 cells. TLR4 and RAGE expression in HMGB1-treated bEnd.3 cells displayed the same pattern of dynamic changes as P-gp. Moreover, TLR4-specific antagonist and RAGE inhibitor could significantly attenuate the effect of HMGB1 on the expression of P-gp, and combined blockage of both TLR4 and RAGE could further decrease this effect. In addition, HMGB1 induced a significant activation of NF-κB p65 in bEnd.3 cells, and this effect could be abolished by the pre-treatment of NF-κB inhibitor SN50. Inhibition of NF-κB could remarkably reduce the overexpression of P-gp in bEnd.3 cells induced by HMGB1, which are in consistent with our previous reports [25, 33]. On the other hand, HMGB1-mediated activation of NF-κB could be significantly inhibited by TLR4 antagonist or RAGE inhibitor. Biological software analysis found there is a potential NF-κB p65 binding site in the promoter sequence of *mdr1a*, and luciferase reporter assay discovered overexpression of p65 in bEnd.3 cells could lead to a significant increase in *mdr1a* promoter activity through the NF-κB binding site. These results suggested that, in bEnd.3 cells, HMGB1 interacted with TLR4 and RAGE, which in turn activated the nuclear translocation of NF-κB, and the activated NF-κB promoted the genetic expression of P-gp directly through the specific binding site (Fig 9).

It is noteworthy that there are some limitations in the present study: 1) P-gp levels in the in vivo experiments may be a consequence of modifications in seizures and may be mediated by some other pathways activated by seizures independently on HMGB1 signaling. For instance, Maroso et al [15] have demonstrated that HMGB1 increased seizure activities in KA-induced mouse model, and seizure-activated pathway- glutamate/COX-2 was shown to be involved in the up-regulation of P-gp in brain capillaries [9, 46]. Thus, there is the possibility that HMGB1 may upregulate P-gp expression through influencing seizure-related pathways such as Glutamate/COX-2 and so on; 2) immortalized brain microvascular cell line, bEnd.3, may not display the fully differentiated phenotype shown by its in vivo counterpart. For example, it was reported to display a less P-gp efflux functionality than the in-vivo microvascular endothelial cells [35]; 3) besides overexpression of P-gp in the primary endothelial barrier of the BBB, the increased P-gp expression in perivascular astrocytes in response to seizures may act as a “second line of defense” for inhibiting the penetration of AEDS to parenchyma [3]. In addition, it has been reported that HMGB1 could also activate TLR4 and RAGE in astrocytes. For this reason, the effect of HMGB1 on the P-gp expression in astrocytes also needs further investigation.

In conclusion, this study indicated that HMGB1 could increase the expression of P-gp in mouse brain endothelial cells via TLR4/NF-κB and RAGE/NF-κB signaling pathways and contributes to the seizure-induced overexpression of P-gp in brain tissues. These experimental findings provided a novel insight into the mechanisms underlying the overexpression of P-gp induced by seizure activities. Pharmacological interventions targeting HMGB1, TLR4, RAGE, and NF-κB may be effective for drug-resistant epilepsy in the future. Currently, various approaches have been explored in order to efficiently inhibit HMGB1 in epileptic brain, including clinical drug glycyrrhizin. In addition, some molecules which directly interact with RAGE or TLR4 as competitive antagonists of HMGB1, such as Box A, S100P-derived small peptides, may also be very promising for the future clinical application.
